# Case Report: A Rare Case of a Ventricular Perivascular Epithelioid Cell Tumor With Histologic Characteristics That Resembled a Primary Cardiac Rhabdomyoma

**DOI:** 10.3389/fcvm.2021.709328

**Published:** 2021-10-25

**Authors:** Jorge Cossío-Aranda, Alberto Aranda-Frausto, Joaquin Berarducci, Nilda Espinola-Zavaleta, Laila González-Melchor, Clara Vázquez-Antona, Gabriela Meléndez-Ramirez, Javier Ivan Armenta-Moreno, Candace Keirns

**Affiliations:** ^1^Instituto Nacional de Cardiología Ignacio Chávez, Mexico City, Mexico; ^2^International Medical Interpreters Association, Boston, MA, United States

**Keywords:** cardiac tumor, PEComa, perivascular epithelioid cell neoplasm, rhabdomyoma of heart, arrhythmia, cardiooncology

## Abstract

We present the case of a young male patient with an initial diagnosis of a rhabdomyoma that was surgically treated at a different hospital when he was 17. After a 2-year disease-free period, the patient presented another intra-cardiac mass. He refused surgical treatment and died 5 years later. Post-mortem immunochemistry studies of both tumors led to the diagnosis of a primary malignant cardiac PEComa with histopathologic characteristics that resembled a rhabdomyoma with abundant “spider cells.”

**Learning Points**

- Cardiac PEComas are very infrequent, and the histological similarities with rhabdomyomas make them a diagnostic challenge.
- It is imperative to correctly identify these tumors since the expectant management that is usually used in rhabdomyomas could lead to the death of these patients.
- There are very few cases of cardiac PEComas in the literature. We encourage clinicians to report their cases so more can be known about this tumor.

## Introduction

Perivascular cell tumors, or PEComas, are mesenchymal neoplasms that are immunoreactive to both smooth muscle and melanocytic markers (1). The PEComa family includes angiomyolipoma, pulmonary clear cell “sugar” tumor, and lymphangioleiomyomatosis. Other tumors with similar features are simply termed PEComas (2). We present a case of a malignant primary PEComa of the heart that was initially confused with a rhabdomyoma.

## Case History

A 19-year-old male presented with sustained ventricular tachycardia (SVT). There was a family history of gastric cancer of unknown type. At the age of 16, the patient had myocarditis that presented with SVT and a mass in the left ventricle that was interpreted as a thrombus. He was treated with immunosuppressants with an adequate clinical response. However, 6 months later, he presented another SVT event and growth of the left ventricular mass to 50 × 30 mm visualized on a transthoracic echocardiogram (TTE). The mass adhered to the interventricular septum, and it was surgically removed when the patient was 17 years old. The histopathologic study performed at the hospital that attended him reported a cardiac rhabdomyoma. After surgery, he was asymptomatic for 2 years.

**Figure 1:**
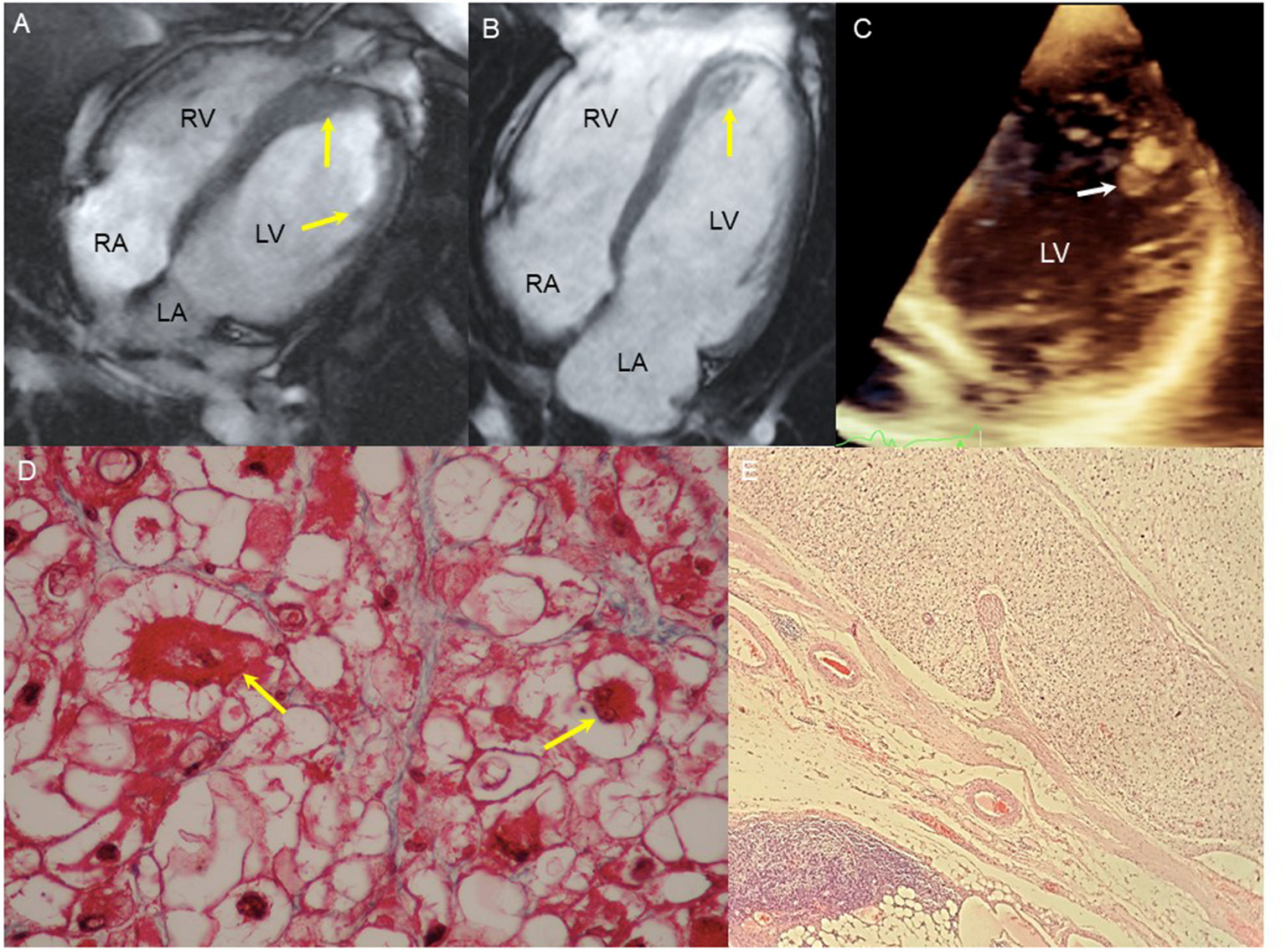
Image and tissue findings. **(A)** CMR 4 chamber image revealing an 8 × 6 mm mass in the lateral LV wall on the papillary muscle insertion and septal thickening (arrows). **(B)** CMR 4 chambers, 36 × 35 mm mass with diffuse enhancement occupying the totality of the LV apex (arrow). **(C)** Four-chamber echocardiogram, 8-mm hyperechoic, homogeneous, rounded mass on the lateral wall of the LV (arrow). **(D)** Micrograph of the first heart tumor. Some nuclear atypia can be seen and two “spider” cells (yellow arrows). Masson trichomic. 40×. **(E)** Micrograph of lymph node with tumoral cells in an alveolar pattern. On the lower left side of the image, the residual lymphatic tissue can be seen. H/E. 4×.

On admission to our institution, a TTE was performed and revealed an 8-mm rounded hyperechoic apical mass that was corroborated by computed tomography (CT) (Figure 1). The patient rejected surgical intervention, and over the following 2 years, sporadic SVT episodes were reported on the 24-h Holter monitoring. On the last consult, the patient described shortness of breath and presented adenopathy in the right axillary region.

Differential diagnosis included rhabdomyosarcoma, PEComa, and primary malignant tumor of unknown origin with metastases.

The final diagnosis of PEComa was reached based on the immunohistochemistry findings (Table 1). Due to the aggressiveness of this tumor, the initial diagnosis of cardiac rhabdomyoma was abandoned, and the possibility of a rhabdomyosarcoma was considered. However, since the first tumor had no characteristics that suggested a rhabdomyosarcoma, and the possibility of a malignant transformation from a rhabdomyoma to a rhabdomyosarcoma had not been reported before, this diagnosis was thoroughly questioned.

**Table 1:** Immunohistochemistry findings.

**Immunohistochemistry Marker**	**First tumor**	**Second tumor**
Vimentin	−	−
HMB-45	+	+
MyoD1	+++	+++
PS-100	+++	+++
Synaptophysin	++	++
Smooth muscle actin	+++	+++
Muscle specific actin	+++	+++
Desmin	−	−
Chromogranin	++	++

*−negative; +mildly positive; ++moderately positive; +++strongly positive*.

## Diagnostic Imaging and Histopathology

**Figure 2:**
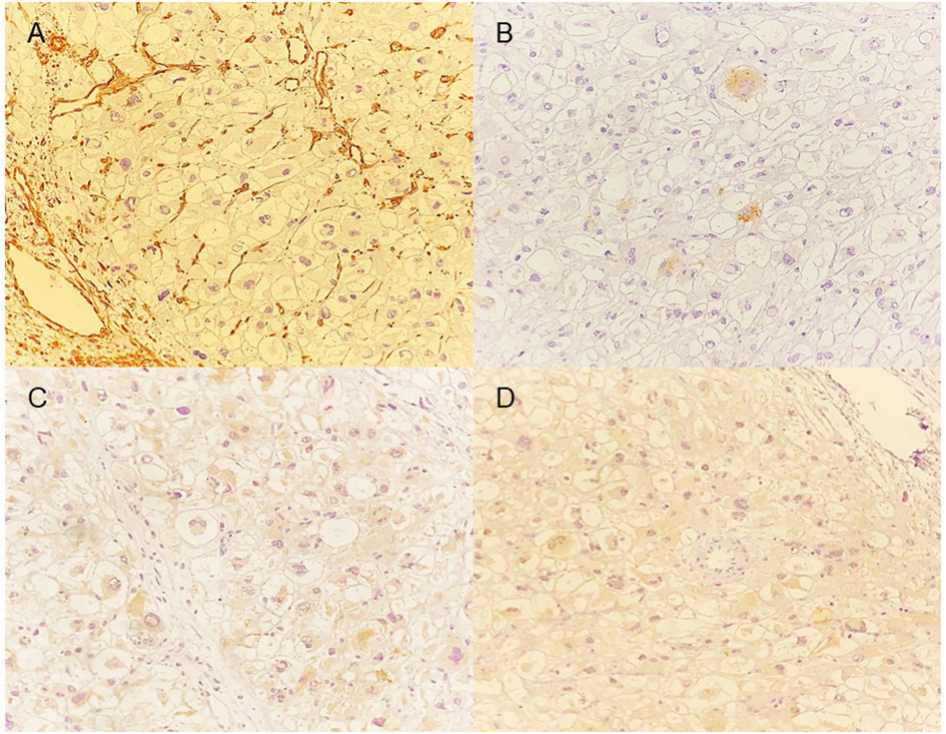
Immunohistochemistry. **(A)** Micrograph of the heart tumor with vimentin that shows negative staining (–): there is no evidence of vimentin in any of the cells. The brown staining in the vessels serves as a control. **(B)** Micrograph of the heart tumor with the HMN-45 marker that shows mild reactivity (+). <25% of the cells show staining. **(C)** Micrograph of the heart tumor that shows moderate reactivity (++) with synaptophysin staining. **(D)** Micrograph of the heart tumor that shows intense positivity (+++) for the MyoD1 marker.

The TEE with 3D remodeling showed an increase in the dimensions of the tumor with occupation of the apex, global hypokinesia, and reduced left ventricular ejection fraction (35%). The CT scan revealed an isodense nodular image of 44 × 26 × 27 mm that was fixed to the deep planes of the internal wall of the anterior thorax along the trajectory of the right internal mammary artery, compatible with regional lymphatic metastasis. In addition, an isodense nodular image of 25 × 20 × 30 mm was observed on the right hepatic lobe with fat interface. The histopathologic study of the axillary nodule showed clear cells with eosinophilic cytoplasm, positive for HMB 45 and SMA and negative for vimentin and desmin (Figure 2).

## Management

The patient declined surgical treatment due to his past surgical failure, and before starting the chemotherapy, he suffered sudden cardiac death.

## Discussion

Due to the tumor's affinity for the vascular walls and the fact that it was immunoreactive for both smooth muscle and melanocytic markers, the definitive diagnosis was established to be an epithelial perivascular cell tumor, or PEComa. To the best of our knowledge, this type of tumor has been reported in other tissues, but only three cases in the heart: two in the ventricles and one in the pericardium (3–5). Since rhabdomyomas are the most frequent primary heart tumors in children (6), and given the morphologic and epidemiologic circumstances, it is understandable that this was the final diagnosis of the other hospital.

PEComas are a group of tumors with a variety of histopathological presentations. Four main groups are described: angiomyolipoma, clear cell “sugar” tumor of the lung, lymphangioleiomyomatosis, and other tumors with similar features that are simply termed PEComa (7). Some PEComas are associated with tuberous sclerosis complexes. The most frequent sites are the gastrointestinal tract, uterus, retroperitoneum, and sometimes soft tissue (3). The cases reported in the literature of malignant PEComas had similar prognoses to our patient with an aggressive behavior and metastatic spread of the tumor.

This case presents an interesting situation involving the previous resection of a heart tumor confirmed by histopathology to be a rhabdomyoma due to its characteristics: ovoid and vacuolated big cells, “spider” cells and positive immunohistochemistry for myoglobin and smooth muscle actin, and negative for desmin and vimentin (Table 2). The immunohistochemistry of this “first” tumor was also positive for SP-100 and negative for vimentin, which should have steered the clinician away from the diagnosis of rhabdomyoma (4). Spider cells are considered a pathognomonic finding of rhabdomyomas (7); this case demonstrates that pathognomonic findings rarely exist. The patient had a 2-year disease-free period, and a subsequent recurrence was documented with metastases to the diaphragm, liver, and regional ganglia. These new findings ruled out the possibility of the primary tumor being a rhabdomyoma, and in conjunction with the immunohistochemical findings, the definitive diagnosis of PEComa was reached.

**Table 2:** Timeline.

**Day 0** 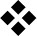 *16-year-old male that presents to the emergency department due to a chief complaint of chest pain, dyspnea, and palpitations*. 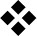 *Electrocardiogram* → Supraventricular tachycardia. 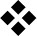 *Trans-thoracic echocardiogram* → Hyperechoic mass in the left ventricle (25 x 21 mm). 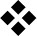 *Cardiac computed tomography* → Hyperdense left intraventricular mass vs. intracavitary thrombus, and findings suggesting myocarditis. 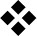 He was scheduled to a heart biopsy due to the high suspicion of a primary heart tumor. **Month 3** 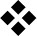 *Heart tumoral biopsy* → Hypertrophy, incipient myocyte degeneration in patch like fashion with fibrosis. 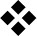 Initiation of immunosuppressive therapy with methylprednisolone and azathioprine due to the diagnosis of myocarditis. **Year 1** 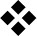 *17-year-old male that presents to the emergency department with severe dyspnea*. 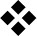 *Electrocardiogram* → Supraventricular tachycardia. 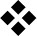 Reinstitution of the immunosuppressive therapy suspended shortly after due to intercostal herpes zoster. 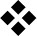 *Trans-thoracic echocardiogram* → Same findings but the mass seems vascularized in the Doppler study. 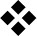 Surgical resection of the ventricular mass (5x3 cm), histologic report concluded the presence of a rhabdomyoma (debated diagnosis, see text). **Year 3** 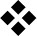 *19-year-old male admitted to the emergency department due to palpitations and chest pain*. 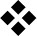 *Electrocardiogram* → Sustained ventricular tachycardia. 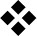 Successful catheter ablation of the arrhythmia. 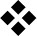 *Trans-esophageal echocardiogram* → Left ventricular rounded, hyperechoic, homogenic 8 mm mass with apical implantation. 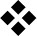 *Cardiac Computed Tomography* → Corroborated the echocardiogram findings with the additional presence of a rounded isodense mass on the anterior papillary muscle. 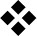 The patient and its family denied surgical intervention. **Year 5** 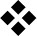 *The patient seeks medical attention because he palpated an axillar mass*. 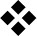 *Biopsy of the axillary ganglionic mass* → Leiomyosarcoma with low grade of malignancy vs. PEComa. 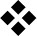 *Trans-thoracic echocardiogram* → Growth of the previous mass. 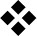 *Cardiac magnetic resonance* → Apical mass with diffuse re-enhancement, 36 x 35 mm, occupying the totality of the left ventricular apex. Same size of the anterior papillary muscle mass. Nodular images on the pectoral muscle and right lobe of the liver. 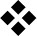 *PET* → Confirmed the metastatic lesions on the anterior wall of the thorax and the liver. 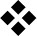 The patient is referred to oncology. 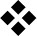 Patient presents sudden cardiac death before the start of the chemotherapy.

The immunohistochemical findings of our patient correlate with those reported in the literature for PEComas. S100 protein was positive in our patient; in our review, we found that one-third of patients present this finding (8).

Biopsy of the axillary ganglia showed characteristics of a malignant alveolar PEComa, with spider cells and a great degree of nuclear atypia. The evidence of nodular involvement diminishes the possibility of a rhabdomyosarcoma, since sarcomas in general rarely involve lymph nodes, and the possibility of a malignant transformation from a rhabdomyoma to a rhabdomyosarcoma has never been reported before to the best of our knowledge.

Cardiac tumors may be symptomatic or found incidentally during evaluation for a seemingly unrelated problem. Symptoms are usually related to their cardiac location, although some can produce systemic symptoms (9). “Malignant” arrhythmias, such as SVT, have been associated with cardiac tumors. In a study of 173 pediatric patients (10) with diverse primary cardiac tumors, SVT was the most prevalent arrhythmia, occurring in 64%. The presence of malignant arrhythmias without an apparent cause should oblige the clinician to investigate the possibility of an undiagnosed cardiac tumor.

## Conclusion

Cardiac primary PEComas are extremely rare, and their histological similarities to rhabdomyomas make them a diagnostic challenge. Although we lack the information to make definitive statements about the prognosis, due to the experience with our patient, we can presume that these are aggressive tumors with a malignant potential. It is essential to report these types of cases to raise awareness that spider cells in the histologic report of a cardiac tumor do not necessarily establish the diagnosis of a cardiac rhabdomyoma.

## Data Availability Statement

The original contributions presented in the study are included in the article/supplementary material, further inquiries can be directed to the corresponding authors.

## Ethics Statement

Written informed consent was obtained from the relevant individual's legal guardian/next of kin, for the publication of any potentially identifiable images or data included in this article.

## Author Contributions

JC-A: Conceptualization (equal), Data curation (equal), Investigation (lead), Methodology (equal), and Writing (equal). AA-F: Conceptualization (equal), Data curation (equal), Investigation (lead), Methodology (equal), and Writing (equal). JB: Conceptualization (lead), Data curation (lead), Investigation (lead), Methodology (equal), and Writing (lead). NE-Z: Conceptualization (equal), Data curation (equal), Investigation (lead), Methodology (equal), and Writing (equal). LG-M: Resources (equal) and Supervision (equal). CV-A: Resources (equal) and Supervision (equal). All authors listed have made a substantial, direct and intellectual contribution to the work, and approved it for publication.

## Conflict of Interest

The authors declare that the research was conducted in the absence of any commercial or financial relationships that could be construed as a potential conflict of interest.

## Publisher's Note

All claims expressed in this article are solely those of the authors and do not necessarily represent those of their affiliated organizations, or those of the publisher, the editors and the reviewers. Any product that may be evaluated in this article, or claim that may be made by its manufacturer, is not guaranteed or endorsed by the publisher.
